# Influence of different concentrations of titanium dioxide and copper oxide nanoparticles on water sorption and solubility of heat‐cured PMMA denture base resin

**DOI:** 10.1002/cre2.527

**Published:** 2022-01-11

**Authors:** Rashin Giti, Maryam Firouzmandi, Neda Zare Khafri, Elham Ansarifard

**Affiliations:** ^1^ Department of Prosthodontics, School of Dentistry Shiraz University of Medical Sciences Shiraz Iran; ^2^ Department of Operative Dentistry, School of Dentistry Shiraz University of Medical Sciences Shiraz Iran; ^3^ Department of Prosthodontics, Student Research Committee, School of Dentistry Shiraz University of Medical Sciences Shiraz Iran; ^4^ Nanobiology and Nanomedicine Research Center Shiraz University of Medical Sciences Shiraz Iran

**Keywords:** denture base, nanoparticles, polymethyl methacrylate, water solubility

## Abstract

**Objectives:**

This study aimed to evaluate the effect of different concentrations of titanium dioxide (TiO_2_) and copper oxide (CuO) nanoparticles on the water sorption and solubility of heat‐cured polymethyl methacrylate (PMMA).

**Materials and Methods:**

Fifty disc‐shaped specimens (10 × 2 mm) of heat‐cured PMMA were prepared and divided into five groups (*n* = 10) to be modified with 2.5 wt.% or 7.5 wt.% of either TiO_2_ or CuO nanoparticles. One group was left unmodified, serving as the control group. Water sorption and solubility were measured by weighing the specimens before and after immersion in distilled water and desiccation. The data were analyzed by using one‐way ANOVA and Tukey's post hoc test (*α* = .05).

**Results:**

The 2.5 wt.% CuO nanoparticles significantly decreased the water sorption (*p* = .016), but did not change the water solubility (*p* = .222) compared with the control group. The 7.5 wt.% CuO and both concentration of TiO_2_ nanoparticles did not change the water sorption, but significantly increased the solubility of heat‐cured PMMA (*p* ≤ .05).

**Conclusion:**

Adding 2.5 wt.% CuO nanoparticles to heat‐cured PMMA decreases the water sorption; although, it has no significant effect on the solubility. Likewise, 2.5 and 7.5 wt.% TiO_2_ and 7.5 wt.% CuO do not affect the water sorption, but increase the water solubility of heat‐cured PMMA.

**Clinical Significance:**

Reinforcing the heat‐cured PMMA denture base resin materials with the right concentration and type of nanoparticles can decrease the water sorption of resin base materials, and consequently can influence the durability of dentures.

## 1 INTRODUCTION

Polymethyl methacrylate (PMMA) acrylic resin has been a common choice for prosthodontics since the beginning of the 20th century. This material has desirable characteristics such as ease of processing and pigmentation, low cost, light weight, stability in the oral cavity, acceptable esthetics, cost‐effectiveness, and minor toxicity (Meng & Latta, 2005; Vojdani et al., 2010). However, some important restrictions are associated with this resin including poor surface and weak mechanical properties like impact and flexural strengths, insufficient ductility, crazing, poor surface hardness, and inadequate antibacterial effect (Murakami et al., 2013).

Acrylic resins absorb water over time mainly due to the polarity of the resin molecules (Lai et al., 2004). Water sorption of acrylic resins in prosthesis acts as a plasticizer and affects the physicochemical and mechanical properties such as the Young's modulus, hardness, transverse strength, and fatigue limit (Barsby, 1992). It also reduces the longevity of a denture within the oral cavity and causes internal stresses that may further lead to cracks and denture fractures (Cucci et al., 1998; Fathi et al., 2019). Solubility represents the mass of the soluble materials from polymers. Soluble materials include initiators, plasticizer, and free monomer. Both water sorption and solubility negatively affect the durability (Phillips, 1991).

To overcome the drawbacks, several attempts have been made to modify and improve the properties of PMMA including various types of fibers and fillers, zirconia, glass fiber, alumina, tin, and copper (Messersmith & Giannelis, 1994). Nanotechnology has recently contributed to the production of different materials (Colvin, 2003). Incorporating the modified nanoparticles of zirconium dioxide (ZrO_2_) into acrylic resin improved the abrasive wear resistance, tensile and fatigue strength, while decreasing the water sorption, solubility and porosity of heat‐cured denture base resin (Mohammed & Mudhaffar, 2012). Jasim and Ismail (2014) found that adding silanized nanoparticles of aluminum oxide (Al_2_O_3_) to PMMA improved the flexural strength of acrylic resin, and decreased the thermal expansion coefficient, water sorption and solubility. Another study showed that adding ZrO_2_ significantly decreased the water sorption and solubility of PMMA (Asar et al., 2013). The properties of polymer nanocomposites depend on the type of incorporated nanoparticles, their size and shape, concentration and interaction with the polymer matrix (Jordan et al., 2005).

Nanoparticles of titanium dioxide (TiO_2_) are among the biocompatible nontoxic materials. These nanoparticles have chemical stability, resistance to corrosion, and high refractive index (Alwan & Alameer, 2015; Emsley, 2001; Ghahremani et al., 2017). TiO_2_ nanoparticles are effective against a wide range of microorganisms such as Gram‐positive and Gram‐negative bacteria, fungi, and viruses (Anehosur et al., 2012). A recent study showed that both 2.5% and 7.5% concentrations of this nanoparticle significantly affect the antimicrobial activity of PMMA denture base material against different species of *Candida* and *Streptococcus* which are mostly presented in the oral cavity (Giti et al., 2021).

Adding 0.5% and 1% TiO_2_ nanoparticles to PMMA were reported to decrease its flexural strength (Sodagar et al., 2013). Another study reported that these nanoparticles reduced the flexural strength without changing the flexural modulus (Hamouda & Beyari, 2014). Yet, improvement of both the flexural strength and Young's modulus was observed following the addition of 0.5% TiO_2_ nanoparticles in another research (Rashahmadi et al., 2017). Alwan and Alameer (2015) noted that adding TiO_2_ nanoparticles to heat‐cured acrylic resin improved the impact strength. Different studies evaluated the mechanical properties of PMMA denture base materials following the addition of different concentrations of TiO_2_ nanoparticles (Hamouda & Beyari, 2014; Rashahmadi et al., 2017; Sodagar et al., 2013); yet, limited information is available about their effects on water sorption and solubility of PMMA denture base material.

Copper oxide (CuO) is another nanoparticle with antimicrobial effects against a wide range of pathogenic bacteria (Karlsson et al., 2008). CuO is more economical and both chemically and physically stable (Chapman et al., 2010). Our previous study showed that both 2.5% and 7.5% concentrations of this nanoparticle had significant influence on the antimicrobial activity of PMMA denture base material against different species of *Candida* and *Streptococcus*. Moreover, increasing the concentration significantly enhanced the antimicrobial effect (Giti et al., 2021).

Despite the imperative effects of water sorption and solubility on durability of denture‐based materials in the oral cavity, to the best of the authors' knowledge, the influence of different concentrations of titanium dioxide and copper oxide nanoparticles on water sorption and solubility of heat‐cured PMMA denture base resin material has not been previously investigated. Thus, the present study aimed to evaluate the effects of adding different concentrations of CuO and TiO_2_ nanoparticles on water sorption and solubility of PMMA denture base material. The null hypothesis was that these nanoparticles would not influence the variables under study.

## 2 MATERIAL AND METHODS

### 2.1 Preparation of specimens

This in vitro study was carried out on 50 disc‐shaped specimens of heat‐cured PMMA (SR Triplex Hot, Ivoclar Vivadent). The specimens were divided into five groups (*n* = 10) to be incorporated with 2.5 or 7.5 wt.% of either TiO_2_ or CuO nanoparticles (2.5% TiO_2_, 7.5% TiO_2,_ 2.5% CuO, and 7.5% CuO). One group was left unmodified, serving as the control group. The concentrations of both nanoparticles were selected according to the previous studies (Naji et al., 2018; Gad et al., 2020; Giti et al., 2021).

To fabricate the PMMA specimens, 10 disc‐shaped wax patterns (10 × 2 mm) were invested with dental stone (Fujirock EP; GC). When the stone set, the flasks (61B Two Flask Compress; Handler Manufacturing) of wax patterns were opened and dewaxed in boiling water for 5 min. The appropriate mass of TiO_2_ nanoparticle powder (average size = 17 nm, 99.9% purity, Fanavaran Daneshgah) and CuO nanoparticles (size = 40 nm, 99.9% purity, Fanavaran Daneshgah) were weighed by using an electronic balance (GR‐300, A&D Company) with accuracy of 0.0001 g, for concentrations of 2.5 and 7.5 wt.%. The powders were mixed with PMMA monomer in aseptic conditions. The suspensions were stirred with an ultrasonic homogenizer to disperse the nanoparticles in the MMA monomer (Gad et al., 2020), and mixed with PMMA powder in liquid:powder volume ratio of 1:3.

Acrylic was packed into the mold spaces. The flask halves were placed into water bath curing unit and processed by being heated to 74°C for 90 min and then to 100°C for 30 min according to the manufacturer's instructions. Once the polymerization process was finished, all the flasks were left to cool down in water, and the specimens were removed after deflasking. Finishing of the specimens was done by using silicon carbide discs (grit 600) and a polishing machine (MetaServ 250 Grinder‐Polisher, Buehler) at 250 rpm, and polishing was done by a cloth wheel and a 0.5‐μm diamond suspension.

### 2.2 Water sorption and solubility test

The specimens were dried in desiccator (Isolab Laborgeräte GmbH) containing freshly dried silica gel (Sigma‐Aldrich), stored in an incubator at 37 ± 2°C. After 24 h, the specimens were removed and weighed to an accuracy of 0.0001 g by using an analytical scale (GR‐300, A&D Company). The 24‐h desiccation cycle was repeated until a constant mass (M_1_) was obtained after 3 days (mass variation was less than ±0.001 mg).

The diameter and thickness of all the specimens were measured by using a digital caliper (Mitutoyo Corp) with an accuracy up to 0.1 mm. The mean diameter was calculated by measuring the diameter of each specimen at two points; these diameters were at right angles to each other. The mean thickness was calculated by measuring the thickness at five equally spaced points on the circumference of the specimen. The volume (V) of each specimen was calculated in mm^3^ according to the following formula: *V* = π(*d*/2)^2^ *h*, where *d* is the diameter and *h* is the thickness of the specimens.

The specimens of each group were immersed in glass vials containing distilled water, wrapped in aluminum foil to exclude light, and placed in an incubator at 37 ± 1°C. The specimens weight was recorded every 24 h until constant weight was achieved (M_2_) after 7 days. Every 24 h, the specimens were removed from the solution, gently wiped with a soft paper towel to remove excess solution, weighed and immediately returned into the solution. At the end of immersion period, the specimens were desiccated as previously mentioned, until the specimens reached the constant mass (M_3_). Water sorption and solubility (µg/mm^3^) were calculated for each of the specimens through the following formulas (Giti et al., 2016):

$$\begin{array}{l} \text{Water sorption}\;=M_{2}-M_{3}/V,\\ \text{Water solubility}\;=M_{1}-M_{3}/V. \end{array}$$

### 2.3 Statistical analysis

Data were analyzed by using SPSS software (IBM SPSS Statistics for Windows, v24.0; IBM Corp). Descriptive data were presented as means and SDs, explored for normality using Shapiro Wilk test. One‐way ANOVA was used to compare water sorption and water solubility among the tested groups. Tukey's post hoc test was used to compare the water sorption and solubility between the groups (*α* = .05).

## 3 RESULTS

**Figure 1:**
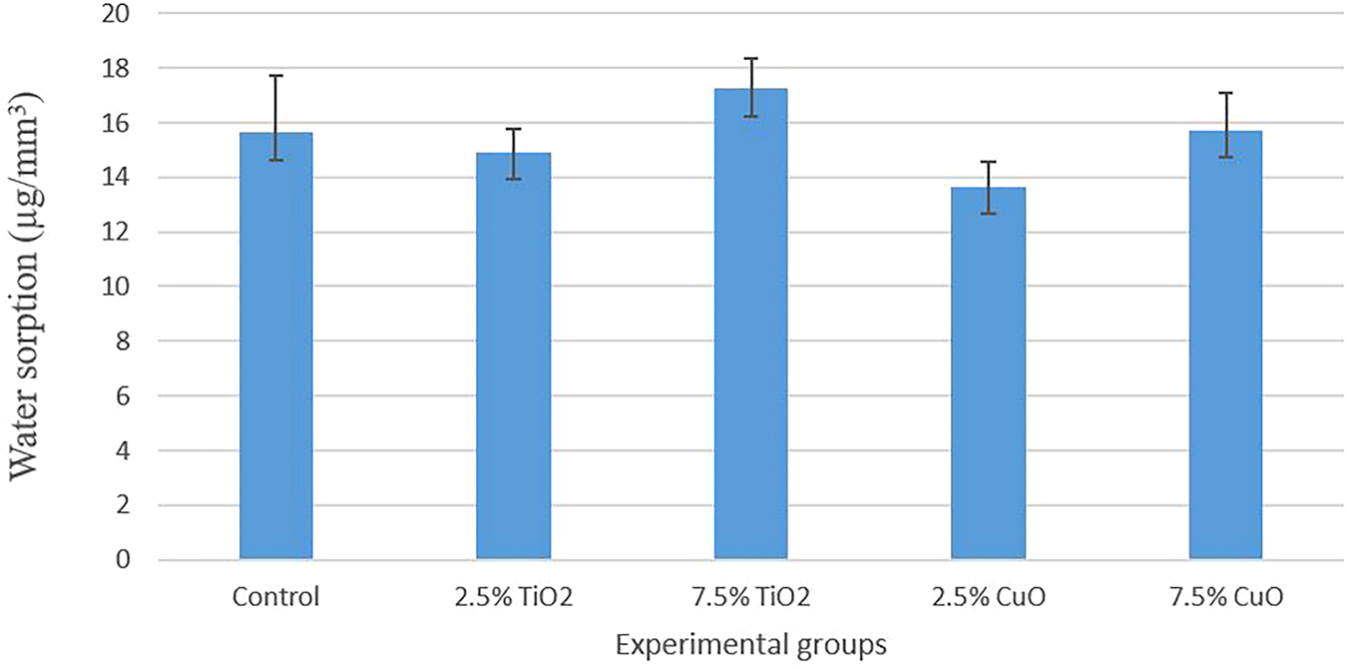
Mean and SDs of water sorption (µg/mm^3^) of experimental groups

**Figure 2:**
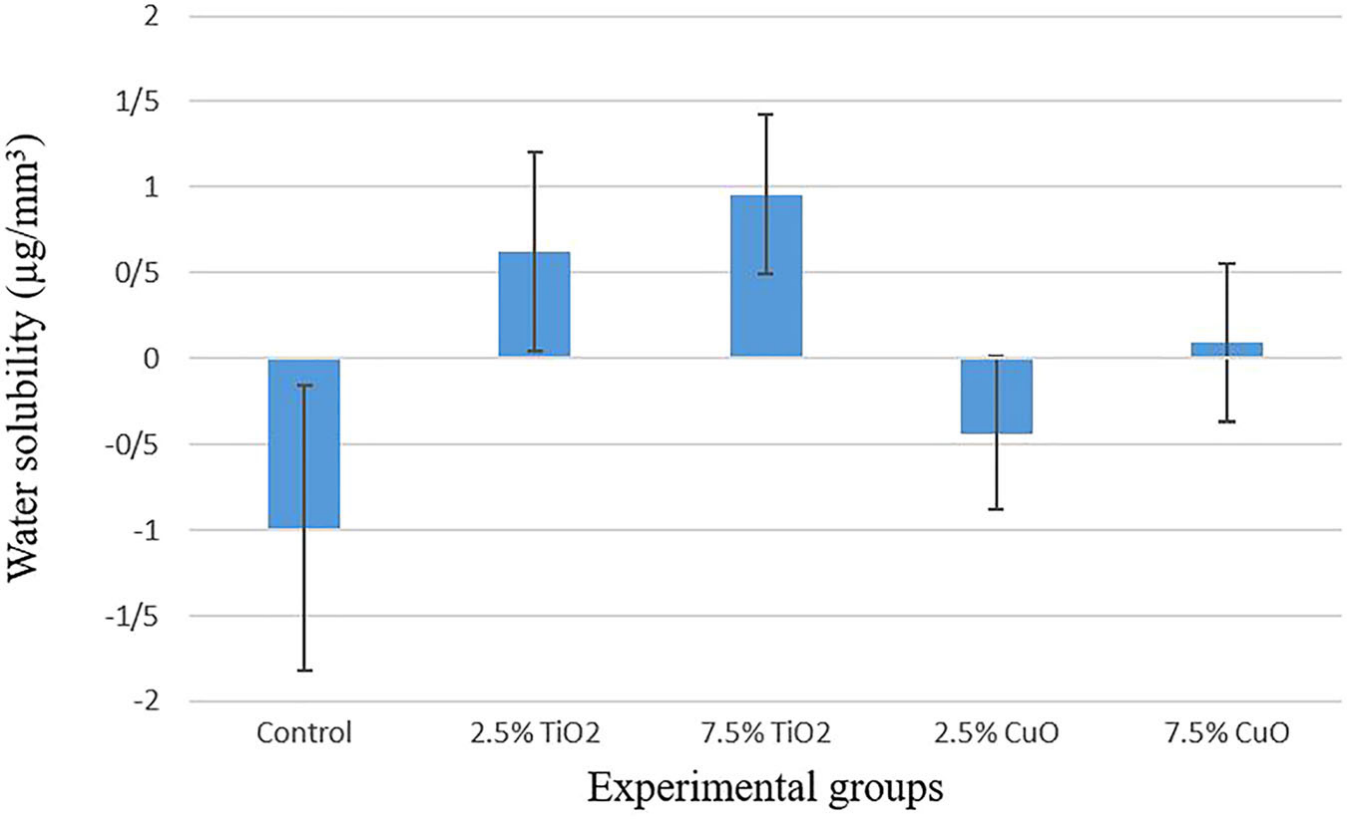
Mean and SDs of water solubility (µg/mm^3^) of experimental groups

According to the results of one‐way ANOVA, the study groups were significantly different in terms of water sorption (*p* < .001) and water solubility (*p* < .001) (Table 1). The mean, SD, and pairwise comparisons between the groups concerning water sorption (Table 2 and Figure 1) and water solubility (Table 3 and Figure 2) were calculated and reported.

**Table 1:** The result of one‐way ANOVA for the water sorption and solubility of the study groups

	Sum of squares	*df*	Mean square	*F*	Sig.
Water sorption					
Between groups	68.405	4	17.101	9.308	0.000
Within groups	82.678	45	1.837		
Total	151.083	49			
Water solubility					
Between groups	24.711	4	6.178	18.628	0.000
Within groups	14.923	45	0.332		
Total	39.634	49			

**Table 2:** Mean, SD, and multiple comparisons of water sorption (µg/mm^3^)

	Concentration		
	0% (control)	2.5%	7.5%
Nanoparticle	Mean ± SD	Mean ± SD	Mean ± SD
TiO_2_	15.63 ± 2.10^ab^	14.91 ± 0.87^aB^	17.23 ± 1.12^bB^
CuO	15.63 ± 2.10^a^	13.64 ± 0.94^bB^	15.71 ± 1.36^aB^

*Note*: Horizontally, different lowercase letters indicate significant differences between different concentrations of each nanoparticle (*p* < .05). Vertically, the same uppercase letters denote no significant difference between different nanoparticles in each concentration (*p* > .05).

**Table 3:** Mean, SD, and multiple comparisons of water solubility (µg/mm^3^)

	Concentration		
	0% (Control)	2.5%	7.5%
Nanoparticle	Mean ± SD	Mean ± SD	Mean ± SD
TiO_2_	−0.98 ± 0.83^a^	0.62 ± 0.58^bA^	0.95 ± 0.46^bA^
CuO	−0.98 ± 0.83^a^	−0.43 ± 0.44^aB^	0.09 ± 0.45^bB^

*Note*: Horizontally, different lowercase letters indicate significant differences between different concentrations of each nanoparticle (*p* < .05). Vertically, different uppercase letters denote significant differences between different nanoparticles in each concentration (*p* < .05).

### 3.1 Water sorption

Tukey's post hoc test showed no significant difference between the experimental groups and the control group (*p* > .05), except for the 2.5 wt.% CuO, which had significantly lower water sorption than the control group (*p* = .016). The findings also indicated that water sorption was concentration‐dependent, as the water sorption of 7.5 wt.% TiO_2_ was significantly higher than that of the 2.5 wt.% TiO_2_ group (*p* = .004), and the water sorption of 7.5 wt.% CuO was significantly higher than that of the 2.5 wt.% CuO group (*p* = .011). No significant difference was detected between CuO and TiO_2_ in neither 7.5 wt.% (*p* = .105) nor 2.5 wt.% concentration (*p* = .236).

### 3.2 Water solubility

The water solubility of both concentrations of TiO_2_ (*p* < .001) and 7.5 wt.% CuO (*p* < .001) were significantly higher than that of the control group. However, the difference was not significant between 2.5 and 7.5 wt.% of TiO_2_ (*p* = .697), nor was it significant between 2.5 and 7.5 wt.% of CuO (*p* = .256). The water solubility of 7.5 wt.% CuO was significantly lower than that of 7.5 wt.% TiO_2_ (*p* = .013) and the water solubility of 2.5 wt.% CuO was significantly lower than that of 2.5 wt.% TiO_2_ (*p* = .001).

## 4 DISCUSSION

The findings rejected the null hypothesis, as the addition of different concentrations of TiO_2_ and CuO nanoparticles affected both water sorption and solubility of PMMA denture base resin. These two features of denture base materials can be affected by several factors like the type of material, amount of plasticizer or filler content, and the solution in which they are immersed (Malacarne et al., 2006). The acrylic resins contain polar carbonyl groups, which control the formation of hydrogen bonds with water and the network topology (Malacarne et al., 2006). Water molecules diffuse between the macromolecules of the material, separate them slightly, and are further transferred to the mass of PMMA and nest between the polymer chains (Saini et al., 2016). Unsaturated bonds of molecules or unbalanced intermolecular forces cause water being absorbed in polymers (Tuna et al., 2008). The attributes of polymer nanocomposites depend on the nature of the added nanoparticles, their size and morphology, concentration, and interactions with the polymer matrix (Kundie et al., 2018). In the present study, water sorption and solubility were measured through the method recommended by the International Organization for Standardization (ISO). Accordingly, the water sorption is the increase in mass per unit volume, and water solubility is the loss of mass from polymers (Miettinen & Vallittu, 1997).

Based on ISO1567:1999, the maximum water sorption and water solubility for heat‐cured PMMA resin should not exceed 32 and 1.6 μg/mm^3^, respectively. Most previous studies obtained water sorption of acrylic resins between 10 and 25 μg/mm^3^ (Ristic & Carr, 1987; Barsby, 1992; Yunus et al., 2005). In the present study, water sorption in all groups ranged between 13.64 and 17.23 μg/mm^3^, and water solubility was between −0.98 and 0.95 µg/mm^3^, meeting the pertinent ISO standards.

### 4.1 PMMA + CuO

The present results showed that 2.5% CuO significantly decreased the water sorption of heat cured‐PMMA. It might be due to the presence of nano‐sized CuO particles in the free spaces between the polymer chains of polymerized PMMA resin. It might have also attracted resin molecules and created more complicated network chains during the curing process, which further eliminated the space for water sorption (Tekale et al., 2019). Another reason could be the replacement of hydrophilic resin with CuO nanoparticles, which decreased the water sorption (Alwan & Alameer, 2015). On the other hand, the present study showed that by increasing the concentration of this nanoparticle to 7.5%, the water sorption of PMMA was increased.

In line with the current findings, Asar et al. (2013) reported significant decrease in the water sorption and solubility of PMMA following addition of various metal oxides including 2% ZrO_2_, 2% TiO_2,_ 2% Al_2_O_3_, and 1% TiO_2_ + 1% ZrO_2_. Similar results were obtained by Jasim et al. (2014) through adding silanized Al_2_O_3_ nanofillers at concentration of 1%, 2%, and 3%. Panyayong et al. (2002) observed that mixtures of titanium dioxide and zirconia at concentrations of 1%, 2%, and 3% decreased the water absorbed by the acrylic resin.

The present study found that 2.5% CuO nanoparticles did not change the water solubility compared with the control group. However, 7.5% CuO notably increased the solubility compared with the control group. The results also revealed that both water sorption and solubility directly depended on the nanoparticles concentration; as the higher concentrations of both nanofillers increased the water sorption and solubility of acrylic resin. The increase of water sorption and solubility by 7.5% CuO might be attributed to the agglomeration of nanoparticles at higher concentrations, which creates a more filler‐filler interaction than the filler–matrix interaction, reducing the intramatrix homogeneity, and adversely affecting the water sorption and solubility of the polymerized material (Mangal et al., 2019).

It has been shown that the CuO nanoparticles increased the thermal stability and enhanced the tensile strength of vinyl‐ester‐based nanocomposites. CuO solubility is highly influenced by the shape of nanostructures (spheres, rods, etc.) (Guo et al., 2007; Laha et al., 2014).

### 4.2 PMMA + TiO_2_

Based on present findings, neither concentrations of TiO_2_ nanoparticles changed the water sorption, but increased the water solubility of heat‐cured PMMA compared to the control group. Chladek et al. (2013) found that increasing the nanosilver concentration increased the sorption and solubility of soft lining material. Kundie et al. (2018) reported that 0.5% and 2% concentrations of alumina micro‐ and nano‐particles slightly increased the water sorption and solubility of PMMA compared with the control group.

The results of the present study contrasted Tekale et al.'s study (2019), which showed that increasing the wt% of 1%, 3%, and 5% silanized TiO_2_ nanoparticles decreased the water sorption of PMMA. Another study documented decreased water sorption and solubility following the addition of 3% wt of treated TiO_2_ nano particles to heat‐cured acrylic resin (Alwan & Alameer, 2015). Acosta‐Torres et al. (2011) detected that PMMA modified with TiO_2_–Fe_2_O_3_ nanocomposites had lower sorption values compared to pure PMMA, despite the similar solubility levels. This could be attributed to the dependence of properties of polymer nano composites on the concentration of nanoparticles, their size and shape, distribution of nanoparticles, and interaction with the polymer matrix (Jordan et al., 2005). Moreover, the interface between a particle and a polymer is sensitive to water due to the high surface energy of the particle. Higher filler concentration increases the particle–polymer interface and consequently higher water sorption and solubility (Panyayong et al., 2002). Accordingly, the significantly higher water solubility values in TiO_2_ group might be mainly related to the increased filler rate and the weak bond to the heat‐cured PMMA matrix.

Among the limitations of this study were the in vitro condition and evaluating only two concentrations of nanoparticles. Future studies are recommended to assess other types and concentrations of nanoparticles in other oral simulating solutions.

## 5 CONCLUSION

(1) The 2.5 wt.% CuO significantly decreased the water sorption and did not change the solubility of heat‐cured PMMA.
(2) Both concentrations of TiO_2_ and 7.5 wt.% CuO nanoparticles significantly increased the water solubility but did not change the water sorption of heat‐cured PMMA than the control group without any nanoparticle.

## CONFLICT OF INTERESTS

The authors declare that there are no conflict of interests.

## AUTHOR CONTRIBUTIONS

Rashin Giti and Elham Ansarifard conceived the idea. Neda Zare Khafri and Maryam Firouzmandi conducted the literature review. Neda Zare Khafri and Rashin Giti performed the experiments. Neda Zare Khafri and Elham Ansarifard collected and analyzed the data. Maryam Firouzmandi and Neda Zare Khafri drafted the paper. Rashin Giti led the writing.

## Data Availability

The date that support the findings of this study are openly available.

## References

[cre2527-bib-0001] Acosta‐Torres, L. S. , López‐Marín, L. M. , Nunez‐Anita, R. E. , Hernández‐Padrón, G. , & Castaño, V. M. (2011). Biocompatible metal‐oxide nanoparticles: Nanotechnology improvement of conventional prosthetic acrylic resins. Journal of Nanomaterials, 2011, 1–8.21808638

[cre2527-bib-0002] Alwan, S. A. , & Alameer, S. S. (2015). The effect of the addition of silanized nano titania fillers on some physical and mechanical properties of heat cured acrylic denture base materials. Journal of Baghdad College of Dentistry, 325, 1–12.

[cre2527-bib-0003] Anehosur, G. V. , Kulkarni, R. , Naik, M. , & Nadiger, R. (2012). Synthesis and determination of antimicrobial activity of visible light activated TiO2 nanoparticles with polymethyl methacrylate denture base resin against Staphylococcus aureus. Journal of Gerontology and Geriatric Research, 1, 1–8.

[cre2527-bib-0004] Asar, N. V. , Albayrak, H. , Korkmaz, T. , & Turkyilmaz, I. (2013). Influence of various metal oxides on mechanical and physical properties of heat‐cured polymethyl methacrylate denture base resins. Journal of Advanced Prosthodontics, 5, 241–247. 24049564 10.4047/jap.2013.5.3.241 PMC3774937

[cre2527-bib-0005] Barsby, M. (1992). A denture base resin with low water absorption. J Dentistry, 20, 240–244. 10.1016/0300-5712(92)90094-s 1430515

[cre2527-bib-0006] Chapman, J. A. , Roberts, W. E. , Eckert, G. J. , Kula, K. S. , & González‐Cabezas, C. (2010). Risk factors for incidence and severity of white spot lesions during treatment with fixed orthodontic appliances. American Journal of Orthodontics and Dentofacial Orthopedics, 138, 188–194. 20691360 10.1016/j.ajodo.2008.10.019

[cre2527-bib-0007] Chladek, G. , Kasperski, J. , Barszczewska‐Rybarek, I. , & Żmudzki, J. (2013). Sorption, solubility, bond strength and hardness of denture soft lining incorporated with silver nanoparticles. International Journal of Molecular Sciences, 14, 563–574. 10.3390/ijms14010563 PMC3565282 23271371

[cre2527-bib-0008] Colvin, V. L. (2003). The potential environmental impact of engineered nanomaterials. Nature Biotechnology, 21, 1166–1170. 10.1038/nbt875 14520401

[cre2527-bib-0009] Cucci, A. L. M. , Vergani, C. E. , Giampaolo, E. T. , & Afonso, M. C. dS. F. (1998). Water sorption, solubility, and bond strength of two autopolymerizing acrylic resins and one heat‐polymerizing acrylic resin. Journal of Prosthetic Dentistry, 80, 434–438. 10.1016/s0022-3913(98)70008-3 9791790

[cre2527-bib-0010] Emsley, J. (2001). Nature's building blocks: An A‐Z guide to the elements. Oxford University Press.

[cre2527-bib-0011] Fathi, A. , Farzin, M. , Giti, R. , & Kalantari, M. H. (2019). Effects of number of firings and veneer thickness on the color and translucency of 2 different zirconia‐based ceramic systems. Journal of Prosthetic Dentistry, 122, 565.e1–565.e7. 10.1016/j.prosdent.2019.08.020 31699449

[cre2527-bib-0012] Gad, M. M. , Abualsaud, R. , Al‐Thobity, A. M. , Baba, N. Z. , & Al‐Harbi, F. A. (2020). Influence of addition of different nanoparticles on the surface properties of poly (methylmethacrylate) denture base material. Journal of Prosthodontics, 29, 422–428. 32233047 10.1111/jopr.13168

[cre2527-bib-0013] Ghahremani, L. , Shirkavand, S. , Akbari, F. , & Sabzikari, N. (2017). Tensile strength and impact strength of color modified acrylic resin reinforced with titanium dioxide nanoparticles. Journal of Clinical and Experimental Dentistry, 9, e661–e665. 28512543 10.4317/jced.53620 PMC5429478

[cre2527-bib-0014] Giti, R. , Vojdani, M. , Abduo, J. , & Bagheri, R. (2016). The comparison of sorption and solubility behavior of four different resin luting cements in different storage media. Journal of Dentistry, 17, 91–97. 27284553 PMC4885678

[cre2527-bib-0015] Giti, R. , Zomorodian, K. , Firouzmandi, M. , Zareshahrabadi, Z. , & Rahmannasab, S. (2021). Antimicrobial activity of thermocycled polymethyl methacrylate resin reinforced with titanium dioxide and copper oxide nanoparticles. International Journal of Dentistry, 2021, 6690806. 33603788 10.1155/2021/6690806 PMC7868146

[cre2527-bib-0016] Guo, Z. , Liang, X. , Pereira, T. , Scaffaro, R. , & Hahn, H. T. (2007). CuO nanoparticle filled vinyl‐ester resin nanocomposites: Fabrication, characterization and property analysis. Composites Science and Technology, 67, 2036–2044.

[cre2527-bib-0017] Hamouda, I. M. , & Beyari, M. M. (2014). Addition of glass fibers and titanium dioxide nanoparticles to the acrylic resin denture base material: Comparative study with the conventional and high impact types. Oral Health and Dental Management, 13, 107–112. 24603926

[cre2527-bib-0018] Jasim, B. S. , & Ismail, I. J. (2014). The effect of silanized alumina nano‐fillers addition on some physical and mechanical properties of heat cured polymethyl methacrylate denture base material. Journal of Baghdad College of Dentistry, 26, 18–23.

[cre2527-bib-0019] Jordan, J. , Jacob, K. I. , Tannenbaum, R. , Sharaf, M. A. , & Jasiuk, I. (2005). Experimental trends in polymer nanocomposites—A review. Materials Science and Engineering: A, 393, 1–11.

[cre2527-bib-0020] Karlsson, H. L. , Cronholm, P. , Gustafsson, J. , & Moller, L. (2008). Copper oxide nanoparticles are highly toxic: A comparison between metal oxide nanoparticles and carbon nanotubes. Chemical Research in Toxicology, 21, 1726–1732. 18710264 10.1021/tx800064j

[cre2527-bib-0021] Kundie, F. , Azhari, C. H. , & Ahmad, Z. A. (2018). Effect of nano‐and micro‐alumina fillers on some properties of poly (methyl methacrylate) denture base composites. Journal of the Serbian Chemical Society, 83, 75–91.

[cre2527-bib-0022] Laha, D. , Pramanik, A. , Laskar, A. , Jana, M. , Pramanik, P. , & Karmakar, P. (2014). Shape‐dependent bactericidal activity of copper oxide nanoparticle mediated by DNA and membrane damage. Materials Research Bulletin, 59, 185–191.

[cre2527-bib-0023] Lai, C. , Tsai, M. , Chen, M. , Chang, H. , & Tay, H. (2004). Morphology and properties of denture acrylic resins cured by microwave energy and conventional water bath. Dental Materials, 20, 133–141. 14706796 10.1016/s0109-5641(03)00084-8

[cre2527-bib-0024] Malacarne, J. , Carvalho, R. M. , Mario, F. , Svizero, N. , Pashley, D. H. , Tay, F. R. , Yiu, C. K. , & de Oliveira Carrilho, M. R. (2006). Water sorption/solubility of dental adhesive resins. Dental Materials, 22, 973–980. 16405987 10.1016/j.dental.2005.11.020

[cre2527-bib-0025] Mangal, U. , Kim, J.‐Y. , Seo, J.‐Y. , Kwon, J.‐S. , & Choi, S.‐H. (2019). Novel poly (methyl methacrylate) containing nanodiamond to improve the mechanical properties and fungal resistance. Materials, 12, 3438–3455. 10.3390/ma12203438 PMC6829541 31640147

[cre2527-bib-0026] Meng, T. R. , & Latta, M. A. (2005). Physical properties of four acrylic denture base resins. Journal of Contemporary Dental Practice, 6, 93–100. 16299611

[cre2527-bib-0027] Messersmith, P. B. , & Giannelis, E. P. (1994). Synthesis and characterization of layered silicate‐epoxy nanocomposites. Chemistry of Materials, 6, 1719–1725.

[cre2527-bib-0028] Miettinen, V. M. , & Vallittu, P. K. (1997). Water sorption and solubility of glass fiber‐reinforced denture polymethyl methacrylate resin. Journal of Prosthetic Dentistry, 77, 531–534. 10.1016/s0022-3913(97)70147-1 9151274

[cre2527-bib-0029] Mohammed, D. , & Mudhaffar, M. (2012). Effect of modified zirconium oxide nano‐fillers addition on some properties of heat cure acrylic denture base material. Journal of Baghdad College of Dentistry, 24, 1–7.

[cre2527-bib-0030] Murakami, N. , Wakabayashi, N. , Matsushima, R. , Kishida, A. , & Igarashi, Y. (2013). Effect of high‐pressure polymerization on mechanical properties of PMMA denture base resin. Journal of the Mechanical Behavior of Biomedical Materials, 20, 98–104. 23455166 10.1016/j.jmbbm.2012.12.011

[cre2527-bib-0031] Naji, S. A. , Behroozibakhsh, M. , Kashi, T. S. J. , Eslami, H. , Masaeli, R. , Mahgoli, H. , Tahriri, M. , Lahiji, M. G. , & Rakhshan, V. (2018). Effects of incorporation of 2.5 and 5 wt% TiO2 nanotubes on fracture toughness, flexural strength, and microhardness of denture base poly methyl methacrylate (PMMA). Journal of Advanced Prosthodontics, 10, 113–121. 29713431 10.4047/jap.2018.10.2.113 PMC5917102

[cre2527-bib-0032] Panyayong, W. , Oshida, Y. , Andres, C. , Barco, T. , Brown, D. , & Hovijitra, S. (2002). Reinforcement of acrylic resins for provisional fixed restorations. Part III: effects of addition of titania and zirconia mixtures on some mechanical and physical properties. Bio‐Medical Materials and Engineering, 12, 353–366. 12652030

[cre2527-bib-0033] Phillips, R. W. (1991). Skinner's science of dental materials. Saunders.

[cre2527-bib-0034] Rashahmadi, S. , Hasanzadeh, R. , & Mosalman, S. (2017). Improving the mechanical properties of poly methyl methacrylate nanocomposites for dentistry applications reinforced with different nanoparticles. Polymer‐Plastics Technology and Engineering, 56, 1730–1740.

[cre2527-bib-0035] Ristic, B. , & Carr, L. (1987). Water sorption by denture acrylic resin and consequent changes in vertical dimension. Journal of Prosthetic Dentistry, 58, 689–693. 10.1016/0022-3913(87)90420-3 3480356

[cre2527-bib-0036] Saini, R. , Kotian, R. , Madhyastha, P. , & Srikant, N. (2016). Comparative study of sorption and solubility of heat‐cure and self‐cure acrylic resins in different solutions. Indian Journal of Dental Research, 27, 288–297. 27411658 10.4103/0970-9290.186234

[cre2527-bib-0037] Sodagar, A. , Bahador, A. , Khalil, S. , Shahroudi, A. S. , & Kassaee, M. Z. (2013). The effect of TiO_2_ and SiO_2_ nanoparticles on flexural strength of poly (methyl methacrylate) acrylic resins. Journal of Prosthodontic Research, 57, 15–19. 23200530 10.1016/j.jpor.2012.05.001

[cre2527-bib-0038] Tekale, R. G. , Mowade, T. K. , & Radke, U. M. (2019). Comparative evaluation of water sorption of heat‐polymerized polymethyl methacrylate denture base resin reinforced with different concentrations of silanized titanium dioxide nanoparticles: An in vitro study. Contemporary Clinical Dentistry, 10, 269–273. 32308289 10.4103/ccd.ccd_499_18 PMC7145259

[cre2527-bib-0039] Tuna, S. H. , Keyf, F. , Gumus, H. O. , & Uzun, C. (2008). The evaluation of water sorption/solubility on various acrylic resins. European Journal of Dentistry, 2, 191–197. 19212546 PMC2635902

[cre2527-bib-0040] Vojdani, M. , Satari, M. , Khajeh Hosseini, S. , & Farzin, M. (2010). Cytotoxicity of resin‐based cleansers: An in vitro study. Iranian Red Crescent Medical Journal, 12, 158–162.

[cre2527-bib-0041] Yunus, N. , Rashid, A. , Azmi, L. , & Abu‐Hassan, M. (2005). Some flexural properties of a nylon denture base polymer. Journal of Oral Rehabilitation, 32, 65–71. 15634304 10.1111/j.1365-2842.2004.01370.x

